# Supplementary material for: Growth Process and Mortality of Sasa borealis Seedlings over Six Years Following Mass Flowering and Factors Influencing Them
Source: Biology (Basel). 2025 May 7;14(5):516. doi: 10.3390/biology14050516 (PMC12109061; doi:10.3390/biology14050516)
Supplement: Supplementary file 1 [file biology-14-00516-s001.zip › biology-3582053-supplementary.pdf]

Supplementary Materials

Growth process and mortality of *Sasa borealis* seedlings over six years following mass flowering and factors influencing them

Hanami Suzuki and Hisashi Kajimura

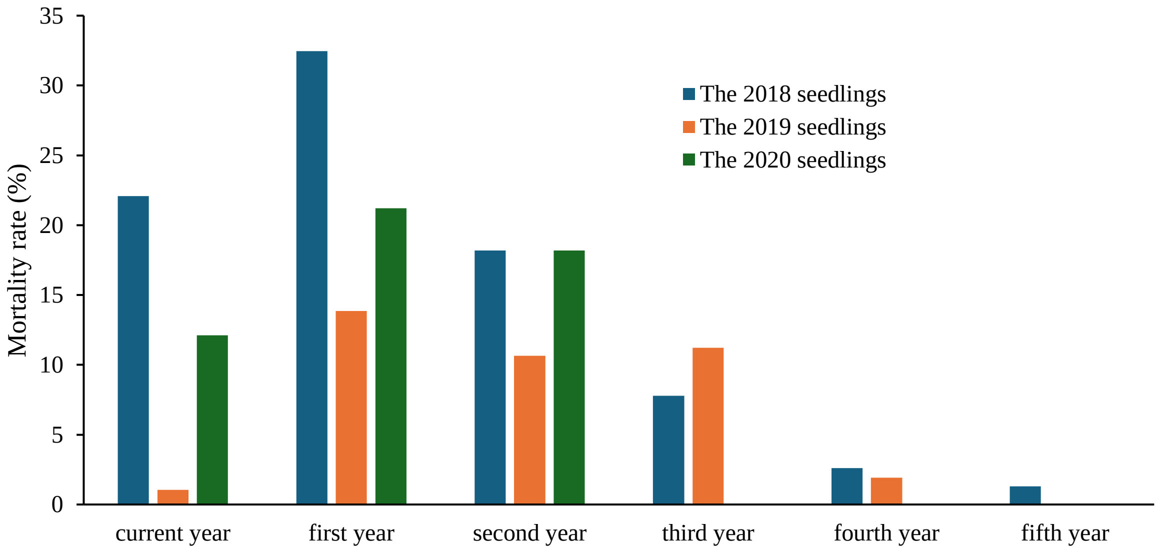

Figure S1
Mortality rates of seedlings germinated in 2018, 2019, and 2020 for each elapsed year.

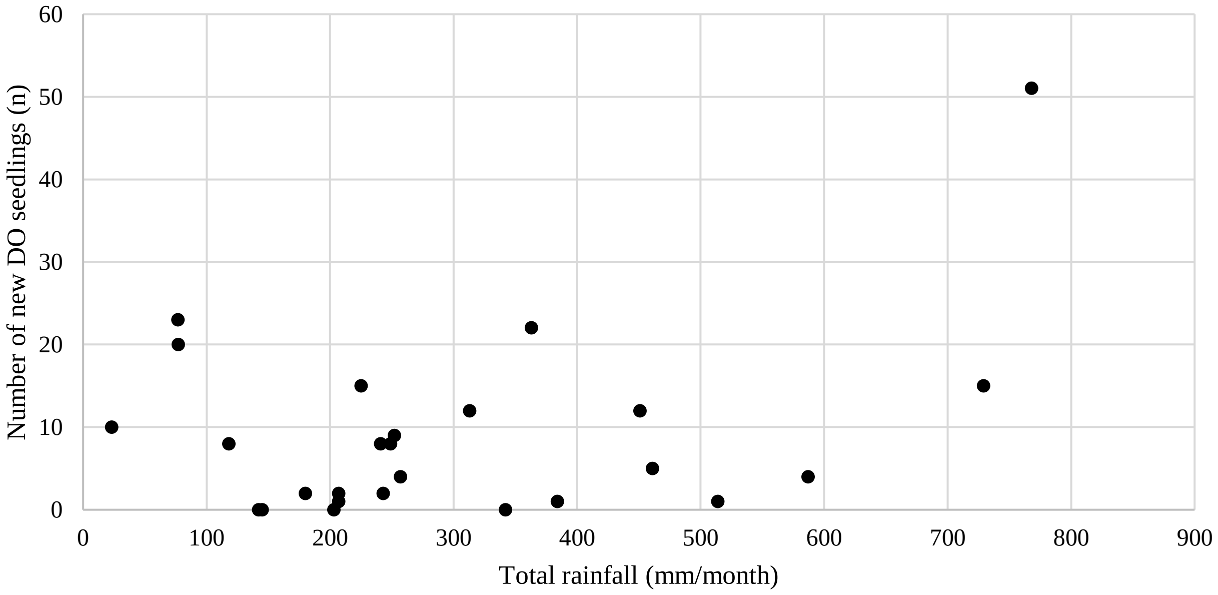

Figure S2
The relationship between the number of new damping off (DO) seedlings and the total rainfall per month. Pearson product-moment correlation coefficient: cor = 0.363, $p = 0.068$, n = 29.

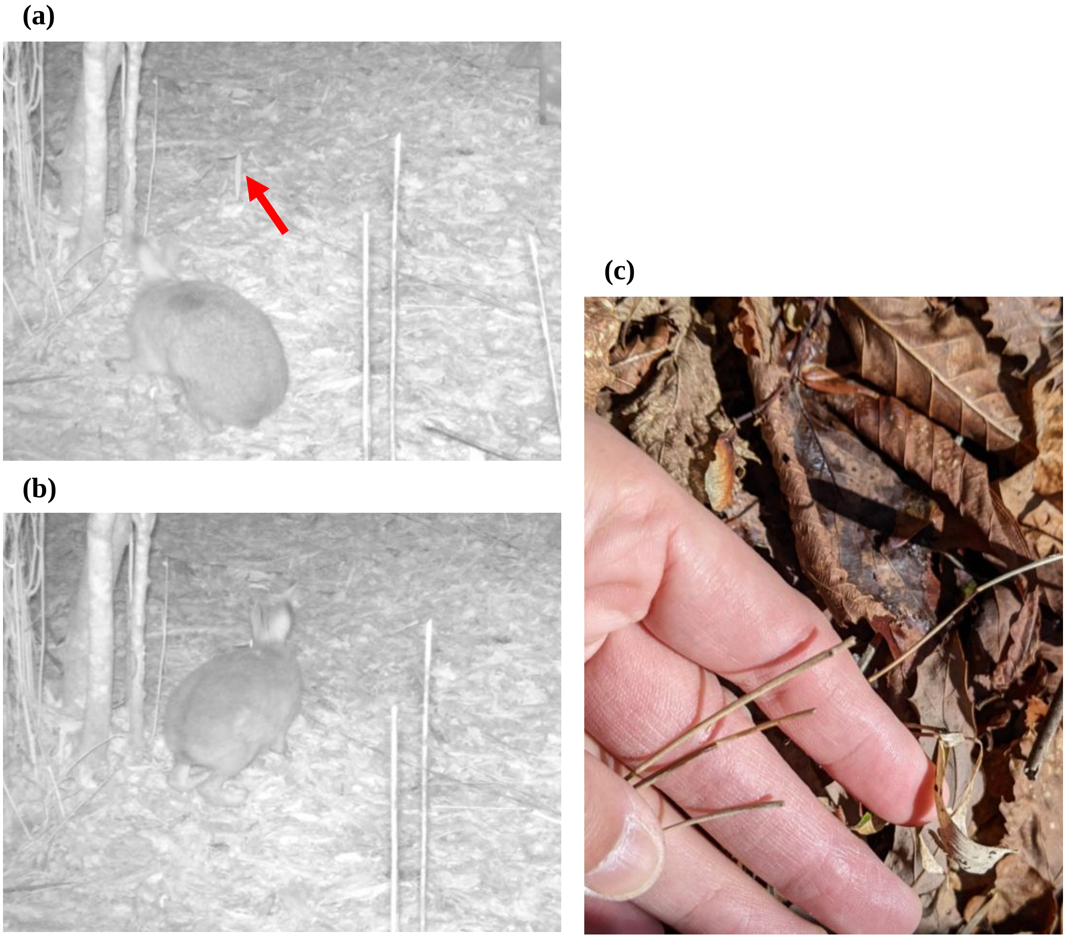

Figure S3
Foraging of seedlings by a hare.
(a) and (b): Hare captured on camera. (c) Stem cut of a seedling indicated by a red arrow in (a).

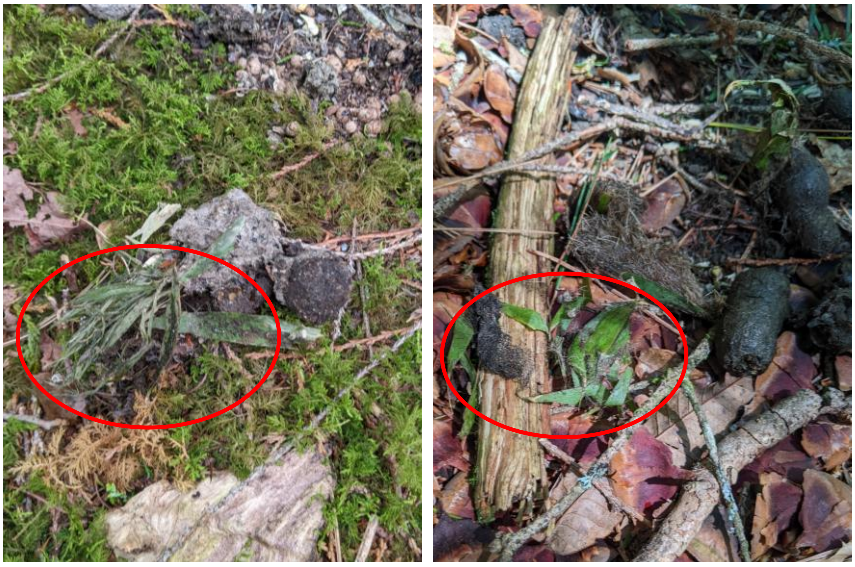

Figure S4
Undigested seedling in raccoon dog feces.

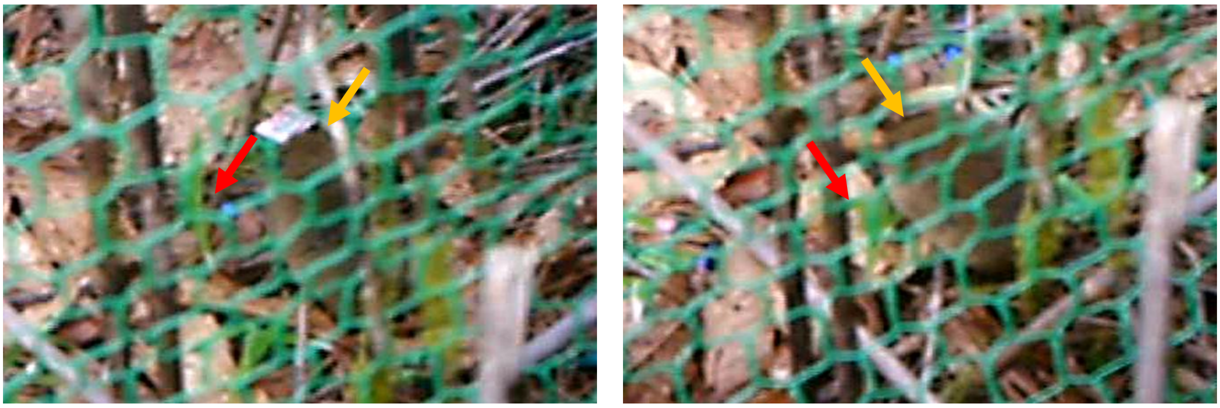

Figure S5
Foraging of seedlings by a field mouse.
Red and yellow arrows indicate a seedling and the mouse, respectively.

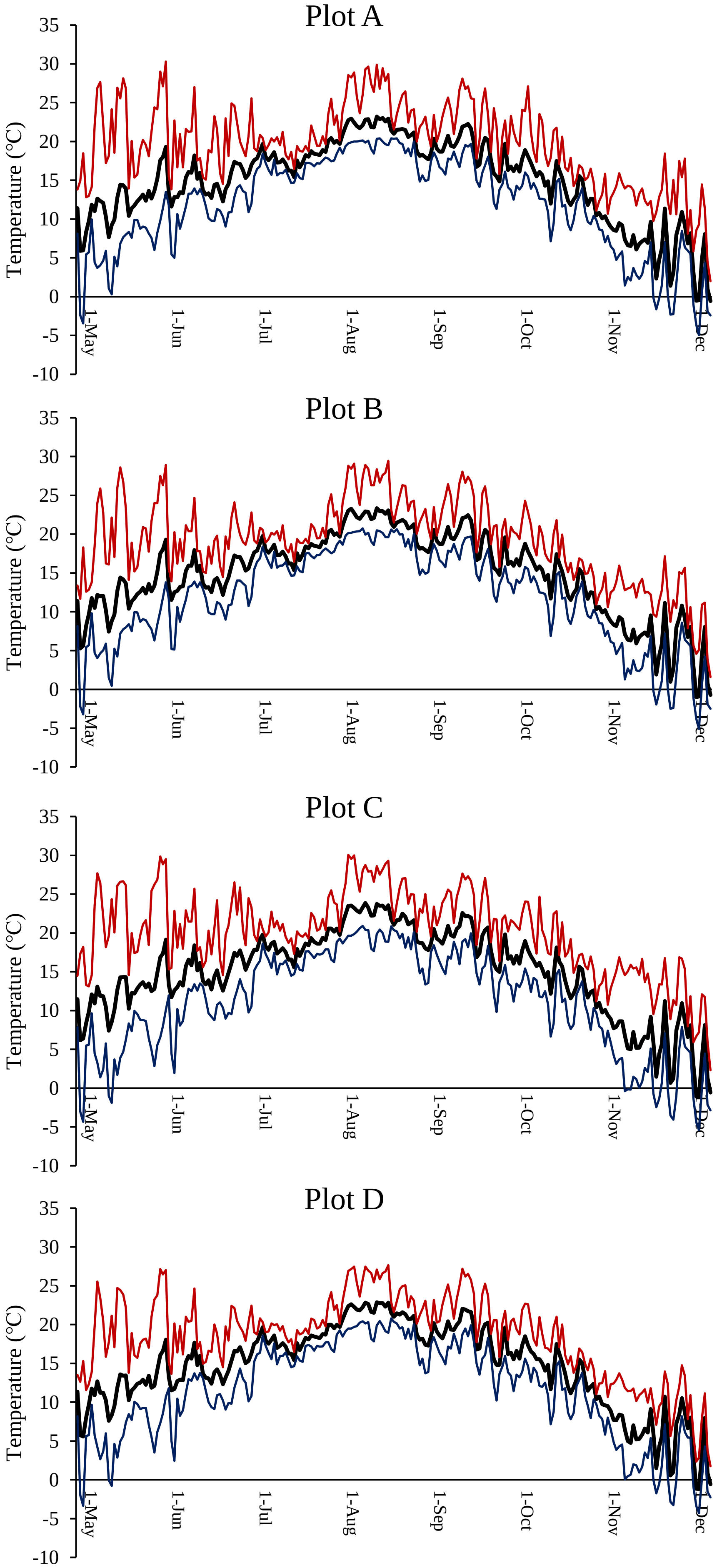

Figure S6

Temperatures in each plot in 2019.

Black, red, and blue lines indicate the average, the maximum, the minimum values for each day, respectively.

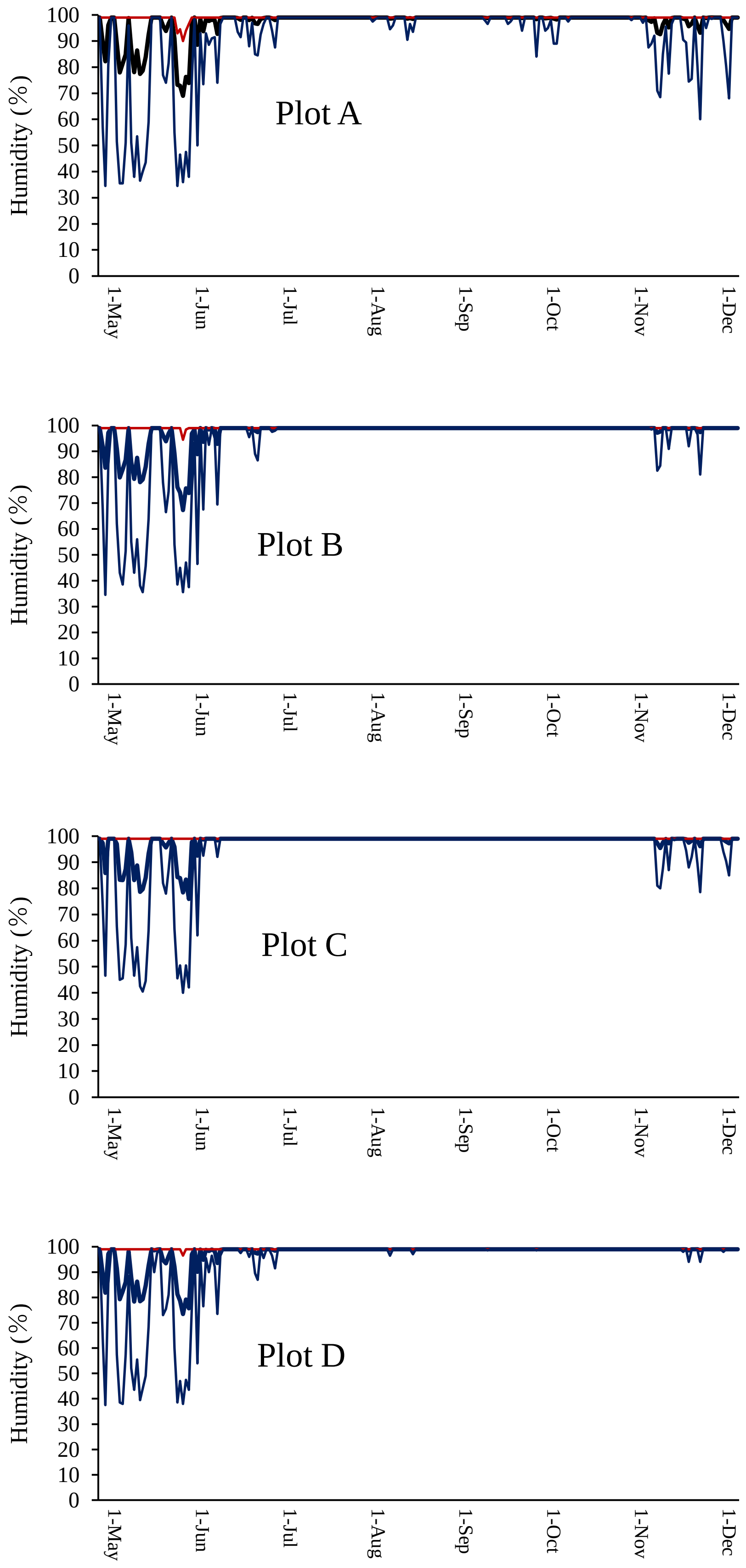

Figure S7
Humidity in each plot in 2019.
Black, red, and blue lines indicate the average, the maximum, the minimum values for each day, respectively.

Table S1
GLM results with the objective variables of the number of seedlings emerged in 2019.
(a) The model within quadrats. (b) The most effective model

(a)

Objective variable	Explanatory variables	Deviance	AIC
Number of emerged seedlings	Total solar radiation+<br>Number of dead culms+<br>Presence of field mice	84.07	212.46
	Total solar radiation	88.77	213.16
	Presence of field mice	88.59	212.98
	Total solar radiation+<br>Number of dead culms	87.66	214.05
	Total solar radiation+<br>Presence of field mice	84.63	211.01
	Number of dead culms+<br>Presence of field mice	86.19	212.58

(b)

Explanatory variables	Estimate	Std. Error	*z*-value	*p*-value
(Intercept)	3.6746	0.15443	23.794	< 2e-16
Total solar radiation	-0.2595	0.13315	-1.949	0.0513
Presence of field mice	-0.1776	0.08659	-2.051	0.0403

Table S2
GLMM results with the objective variables of (a) culm height in each year and (b) culm height grown during one year.

	Explanatory variables	Estimate	Std. Error	*t*-value	*p*-value
**(a)**	(Intercept)	-1.4070	0.0201	-69.941	< 2e-16***
	Solar radiation	0.0029	0.0029	0.986	0.3243
	Temp_ave.	-0.0042	0.0008	-5.006	5.56E-07***
	Temp_max.	-0.0080	0.0002	-32.641	< 2e-16***
	Temp_min.	-0.0020	0.0011	-1.835	0.0666
	Humidity	0.0152	0.0000	499.253	< 2e-16***
	Numver of seedlings_pre.	0.0016	0.0003	5.368	7.95E-08***
	Numver of seedlings_cur.	-0.0022	0.0003	-6.771	1.28E-11***
	Soil_moisture	0.0010	0.0002	4.509	6.50E-06***
	Soil_EC	0.0091	0.0228	0.399	0.6897
	Plot	0.0032	0.0054	0.602	0.5471
	Quadrat	0.0018	0.0043	0.429	0.6676
	Snow_days	-0.0030	0.0001	-58.896	< 2e-16***
	Snow_depth	0.0042	0.0001	30.737	< 2e-16***
	Precipitation	0.0014	0.0000	122.88	< 2e-16***
**(b)**	(Intercept)	263.5062	0.0052	51157.54	< 2e-16***
	Solar radiation	-0.0430	0.0050	-8.515	< 2e-16***
	Temp_ave.	-4.1071	0.0051	-803.465	< 2e-16***
	Temp_max.	0.1253	0.0051	24.567	< 2e-16***
	Temp_min.	-3.9293	0.0049	-794.968	< 2e-16***
	Humidity	-1.1235	0.0048	-232.593	< 2e-16***
	Numver of seedlings_pre.	0.0152	0.0025	5.962	2.50e-09***
	Numver of seedlings_cur.	-0.0193	0.0029	-6.689	2.25e-11***
	Soil_moisture	0.0056	0.0024	2.336	0.0195*
	Soil_EC	0.3994	0.0051	77.626	< 2e-16***
	Plot	0.4103	0.0053	78.101	< 2e-16***
	Quadrat	0.1118	0.0052	21.43	< 2e-16***
	Snow_days	0.7297	0.0025	289.874	< 2e-16***
	Snow_depth	-0.3716	0.0046	-81.055	< 2e-16***
	Precipitation	-0.4646	0.0023	-201.581	< 2e-16***
	Years	-4.7074	0.0050	-933.009	< 2e-16***

Table S3

Deer foraging targets captured on camera

	Mature individual of *Sasa borealis*	Seedling of *S. borealis*	Leaf of trees	Fallen leaf	Seedling of tree
7-Aug ~ 30-Nov-2018	0	0	0	0	0
1-Dec-2018 ~ 30-Nov-2019	0	0	6	0	0
1-Dec-2019 ~ 30-Nov-2020	1	0	2	0	0
1-Dec-2020 ~ 30-Nov-2021	0	0	2	0	0
1-Dec-2021 ~ 30-Nov-2022	0	0	0	0	1
1-Dec-2022 ~ 29-Nov-2023	0	0	4	3	1

Table S4

GLM results with the objective variables of presence or absence of foraging damage.

	**Culm height**				**The number of culms**				**The number of leaves**			
	**Estimate**	**Std. Error**	***t*-value**	***p*-value**	**Estimate**	**Std. Error**	***t*-value**	***p*-value**	**Estimate**	**Std. Error**	***t*-value**	***p*-value**
(Intercept)	0.7890	0.2964	2.662	0.0079**	0.9530	0.1080	8.823	< 2e-16 ***	6.5201	0.4743	13.747	< 2e-16 ***
Culm_before	0.0271	0.2516	0.108	0.9141	-0.3333	0.0883	-3.773	0.0002***	-0.1807	0.3879	-0.466	0.6414
Leaf_before	0.3598	0.2414	1.490	0.1365	0.1951	0.0880	2.218	0.0268*	0.2360	0.3863	0.611	0.5414
Culm_current	-2.0210	0.2022	-9.994	< 2e-16 ***	-0.2787	0.0719	-3.878	0.0001***	-1.0604	0.3155	-3.361	0.0008***
Leaf_current	0.5146	0.3115	1.652	0.0989.	0.3361	0.1162	2.893	0.0039**	0.4807	0.5101	0.942	0.3463
Years	0.1141	0.1030	1.108	0.2681	-0.0009	0.0374	-0.024	0.9805	-1.9626	0.1641	-11.962	< 2e-16 ***
Culm_before: Leaf_before	-0.0880	0.5684	-0.155	0.8770	0.1031	0.1889	0.546	0.5853	-0.7755	0.8293	-0.935	0.3499
Culm_current: Leaf_current	0.9465	0.7742	1.223	0.2218	-0.2304	0.2925	-0.788	0.4310	-1.6023	1.2842	-1.248	0.2124

The presence or absence of foraging damage was used as a dummy variable (absence, 0; presence, 1). *: $p < 0.05$, **: $p < 0.01$, ***: $p < 0 .001$
